# Jail Diversion Program Implementation in a Homeless Patient With Schizophrenia: A Case Report

**DOI:** 10.7759/cureus.31567

**Published:** 2022-11-16

**Authors:** Lauren M Katzell, Sophia Array, Colleen Bell, Ana Turner

**Affiliations:** 1 Psychiatry, University of Florida College of Medicine, Gainesville, USA; 2 Psychiatry, University of Florida Health Jacksonville, Jacksonville, USA; 3 Psychiatry, Sulzbacher Center, Jacksonville, USA

**Keywords:** economic costs, correctional facilities, equity, community psychiatry, intellectual disability, schizophrenia, recidivism, jail diversion, homeless persons, case report

## Abstract

With a majority of prisoners in the United States having a history of mental illness and only half of these receiving mental health treatment, it became imperative to develop programs to assist those with mental illness to avoid repeated incarceration. The Eleventh Judicial Circuit Criminal Mental Health Project (CMHP) was developed to decrease recidivism in patients with serious mental illness (SMI). This case details how the Mental Health Offender Program (MHOP), created following the CMHP model, allows for substantial decreases in costs associated with non-violent offenders with SMI and provides wraparound services to improve their functioning and quality of life.

## Introduction

With the rate of mental disorders ranging from 3%-12% higher in the incarcerated population compared to that of the general population, jailed persons must receive mental healthcare [1]. According to Abramsky and Fellner, there are three times more individuals with severe mental illness (SMI) in prison than in psychiatric hospitals in the United States [2]. Additionally, the relative annual risk of a patient with SMI being jailed is 150% greater than that of being admitted to a hospital for inpatient psychiatric care [3]. However, jails in the United States notoriously do not provide adequate mental healthcare and are associated with worsening psychiatric symptoms [4,5]. This leads to a high rate of recidivism, especially for non-violent offenders suffering from SMI, which is associated with significant community costs [6,7].

To address this issue in Miami, Florida, the Eleventh Judicial Circuit Criminal Mental Health Project (CMHP) was created to divert individuals with SMI from jail into comprehensive treatment and support services [8]. This program proved successful, resulting in an annual cost avoidance of $12,000,000 and a 45% lower jail population [9]. Using this template, the Mental Health Offender Program (MHOP) was started in Jacksonville, Florida, with hopes to find similar results in a different part of the state. The MHOP works to improve mental healthcare and social services for individuals with SMI, usually schizophrenia or schizoaffective disorder, by diverting non-violent offenders, primarily those with only misdemeanors, from the criminal justice system. These individuals are seen by a psychiatrist at regular intervals and started on long-acting injectable anti-psychotic medications to improve medication adherence and reduce loss of follow-up in this vulnerable population.

Criteria for selection into MHOP include having a diagnosis of SMI, being arrested four or more times since 2017, no sexual offender classification, no open felony cases, and not currently on felony probation or parole. Through the MHOP program, the Sulzbacher Center in Jacksonville, Florida, provides the defender with case management, psychiatric and medical treatment, therapy, housing assistance, and other wraparound services. Cases are reviewed every two to six weeks, depending on client stability, and those who successfully complete the program have their case dismissed as part of the deferred prosecution agreement [10]. Here we present a case of a patient with SMI and subsequent substantial community cost who was diverted from jail through admission to the MHOP program. Written informed consent for this case report was obtained from the patient.

## Case presentation

Mr. X is a 35-year-old male who was chronically experiencing homelessness. He was initially encountered by Sulzbacher staff on outreach. The patient initially was not agreeable to speaking with staff; however, when he was given food, he slowly began to speak to staff while eating. He was distractable and attempted to walk away during the interview, but he was able to be redirected. Staff at Sulzbacher Center in Jacksonville, Florida, were aware of Mr. X and noted that he often had trouble with self-care and lost the belts, shoes, and clothing they provided him soon after receiving them. He was previously seen at the Mental Health Resource Center (MHRC) in Jacksonville, Florida, which provides outpatient, inpatient, and rehabilitative mental health services, but he was unable to state other psychiatric histories or past diagnoses. He stated he would be willing to meet with the Supplemental Security Income/Social Security Disability Insurance Outreach, Access, and Recovery (SOAR) processor. 

Mr. X presented to the shelter initially shoeless, nonverbal, covered in his own feces, unaware of the cold or elements, floridly psychotic, and sleeping in dumpsters. Shelters previously refused him due to difficulty toileting. Staff at other shelters noted that he would routinely have feces smeared on his hands and cause flooding from the toilets. This led to immense difficulty with housing. Mr. X was arrested 97 times since 2017, with arrests consisting of non-violent offenses, mainly trespassing and loitering. He also had countless hospitalizations upon review of medical records. From just one hospital in the area, the University of Florida Health Jacksonville, Mr. X had 68 emergency department admissions, eight inpatient psychiatric admissions, and one inpatient medicine admission since 2017. He also had 95 admissions to MHRC in that same time frame (Table 1).

**Table 1:** Pre-enrollment statistics

Arrests	97
Emergency Department Admissions	68
Inpatient Psychiatric Admissions	8
Inpatient Medicine Admission	1
Mental Health Resource Center Admissions	95

On initial mental status exam, the patient appeared disheveled and underweight with soiled clothing. His behavior was impulsive, and he made poor eye contact. He had impoverished speech and was not responding to internal stimuli at the time of the interview. He was disoriented to situation and exhibited impaired concentration and attention. He stated his mood was “fine” and exhibited a restricted, flat affect. His insight and judgment were impaired. He exhibited thought blocking and did not endorse suicidal or homicidal ideation. His motor activity was grossly normal.

The differential diagnosis for Mr. X included primary psychotic disorder (i.e., schizophrenia or brief psychotic disorder), major depressive disorder with psychotic features, unspecified personality disorder, substance-induced psychosis, psychosis secondary to organic causes such as electrolyte abnormality or infection, major neurocognitive disorder, and delirium.

After further examination, Mr. X was diagnosed with schizophrenia, intellectual disability, and tobacco use disorder. He had previously been started on oral risperidone 2mg daily and, at MHRC, was prescribed haloperidol decanoate 50mg intramuscularly monthly. At his follow-up visit approximately one month later, he exhibited continued bizarre behavior, disorganized thoughts, impoverished speech, significant impairment in social interactions, and negative symptoms. He did not appear to benefit from haloperidol decanoate and refused to take pills by mouth. Thus, he was prescribed paliperidone palmitate 234mg intramuscular injection monthly.

He stated that he thought the shot, paliperidone palmitate, was helping him, and he wanted to stay on it. He said it was helping with the voices he was hearing but did not elaborate further. He was able to sit still and answer questions, asked for snacks, and put on a mask when prompted. He was started on haloperidol decanoate 50mg monthly in addition to the paliperidone palmitate due to continued disorganized behaviors and psychotic symptoms. The haloperidol decanoate was subsequently increased to 100mg. He was switched to paliperidone palmitate 819mg every three months after three months of paliperidone palmitate therapy. He was also started on trazodone 50mg oral nightly for insomnia, which was eventually increased to 100mg. On this regimen, Mr. X showed notable improvement but still had thought-blocking and bizarre behaviors at times.

In an effort to get Mr. X housing, the staff was working on behavior modification for bathroom use. It was found that when Mr. X was going to the bathroom, he was wrapping the entire roll of toilet paper around his hand and placing it in the toilet. When he flushed the toilet, this caused repeated flooding. He was also having trouble wiping his hands and not washing his hands. This led to him having feces on his person as well as smearing it on surfaces, leading to improper sanitation in public facilities. To address these behaviors, his psychiatrist added 5mg of oral haloperidol twice daily in addition to his haloperidol decanoate. More importantly, the team worked with him on managing toileting behaviors, redirecting him to use certain bathrooms that had the paper products from the bathrooms removed, with staff readily on hand to provide him with a limited supply of paper products sufficient for single use. With Mr. X now staying in the shelter, he was able to have special staff assigned to him to assist with taking oral medication and assist with basic care, toileting, and nursing, with additional staff hired to assist him with evenings and weekends. With control of toileting behaviors, he was able to stay in a homeless shelter and ultimately apply for an assisted living facility. During the day, he began attending a day program at the local Community Resource Center. 

At his follow-up visit, he stated he enjoyed the psychosocial rehabilitation program he was attending and denied auditory or visual hallucinations but was responding and laughing inappropriately during his session. At that visit, he was shaven with a recent haircut. His clothes were slightly disheveled, but he was wearing shoes, a shirt, pants, and a belt appropriately. He avoided eye contact. His behavior was slightly distractable, but he had no freezing periods. He was responding to internal stimuli. His speech was impoverished, but he was more responsive than at prior visits. He stated that his mood was good. His affect was restricted. His thought process was concrete, though with less thought blocking than at prior visits, and he was able to form complete sentences. He exhibited no suicidal or homicidal ideation or delusions.

As Mr. X now had stable housing, the decision was made to start him on clozapine. His oral haloperidol and haloperidol decanoate were discontinued. His absolute neutrophil count was checked and was normal. He was started on 25mg by mouth daily for one week and then increased to 50mg daily for one week. He was scheduled for weekly complete blood counts for the first six months. He was agreeable to this plan. At subsequent follow-up visits, his clozapine was uptitrated to 300mg by mouth nightly. He was placed in an assisted living facility. His Trinza was discontinued, and his clozapine was switched to the morning.

**Figure 1:**
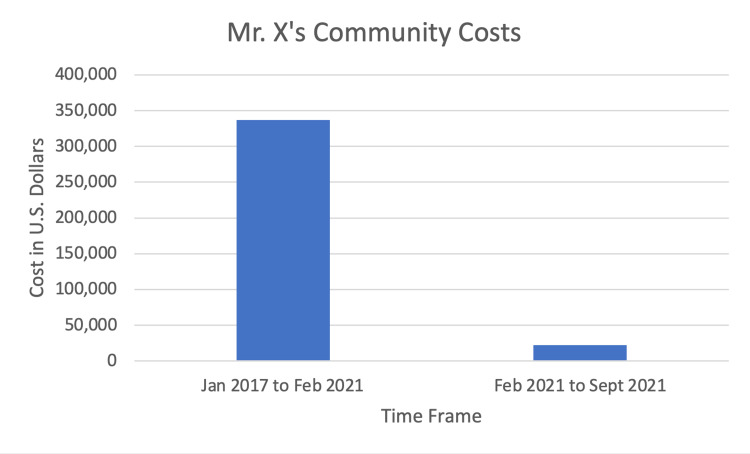
Mr. X's community costs, factoring in funding for the pilot program

Eventually, Mr. X was transitioned back to a combination of haloperidol decanoate and clozapine, on which he exhibited increased control of his psychiatric and behavioral symptoms. He was able to receive an income, and guardianship proceedings were started. He was transitioned to monthly labs for clozapine. Community costs for Mr. X were calculated to include jail bookings, days in jail, psychiatric hospital evaluations when sent directly from jail, and subsequent hospital stays. Mr. X’s community costs decreased from $337,008 from January 2017 to February 2021 before enrollment in MHOP to $22,147 during the pilot period of nine months from 2/1/2021 to 9/30/21, factoring in funding from the city and sheriff's office that was utilized to fund medications, housing costs, and other social services (Figure 1). His self-reported quality of life also improved dramatically.

## Discussion

The services Mr. X required initially included shelter, clothing, food, water, hygiene items, money/income, food stamps, transportation, including bus passes, phone and internet access, medical attention, and medication. This required a multidisciplinary approach with a team of trained individuals to monitor Mr. X throughout the day until he was transitioned to his assisted living facility. This also required hiring additional staff to assist him on evenings and weekends when MHOP staff was not on campus.

Dual antipsychotics, haloperidol plus paliperidone, were originally used due to the failure of multiple monotherapies and the inability to follow up for clozapine labs. However, when the patient was able to tolerate the oral medication and find stable housing with supervision, he was able to have assistance with taking oral medications, and the street psychiatry team drew his clozapine monitoring labs at his day program. His final regimen was a combination of haloperidol decanoate and clozapine, as this seemed to best control his psychotic symptoms and address the behavioral challenges, allowing him to remain in his assisted living facility.

The MHOP pilot program, which consisted of 20 participants and ran from 2/1/21 to 9/30/21, resulted in 86.7% of defendants in permanent housing, with 13.3% in temporary housing while awaiting a permanent home. After the pilot, 73.3% were receiving disability benefits, and 26.7% of patients had benefits pending. The total cost for the 20 pilot participants in 2020 was $362,218 and in 2021, before entry into MHOP was $57,748. After entry into MHOP, the community costs were $12,631. The monthly average arrest rate dropped 81% for pilot participants. Interestingly, only 70% of eligible individuals agreed to participate in this program, despite being told they would receive housing, help with an application for benefits, healthcare treatment, clothing, and food [10].

## Conclusions

Mr. X’s case demonstrates the necessity of wraparound services and dedicated staff to develop individualized treatment plans for non-violent offenders with SMI. Repeated incarceration not only worsens mental illness in this population but also leads to increased costs in comparison to providing support that can treat mental health crises, reduce jail time, and improve quality of life. While these extensive services through comprehensive organized programs are not available nationwide, this case serves to demonstrate the efficacy and cost savings of such initiatives. In areas where there are disproportionate numbers of inmates with SMI and repeat incarcerations for non-violent crimes, the creation of jail diversion programs should be considered.
